# Occurrence and Phylogenetic Analysis of Zoonotic Enteropathogenic Protist Parasites in Asymptomatic Domestic Ruminants from Portugal

**DOI:** 10.3390/pathogens12111341

**Published:** 2023-11-11

**Authors:** Sara Gomes-Gonçalves, Josman Dantas Palmeira, Helena Ferreira, Sérgio Santos-Silva, João R. Mesquita

**Affiliations:** 1 Department of Biology, University of Aveiro, Campus de Santiago, 3810-193 Aveiro, Portugal; saragomes2000@hotmail.com; 2 UCIBIO—Applied Molecular Biosciences Unit, University of Porto, 4050-313 Porto, Portugal; josmandantasp@gmail.com (J.D.P.); hferr@ff.up.pt (H.F.); 3 Associate Laboratory i4HB, Institute for Health and Bioeconomy, University of Porto, 4050-313 Porto, Portugal; 4 Microbiology, Biological Sciences Department, Faculty of Pharmacy of University of Porto, 4050-313 Porto, Portugal; 5 School of Medicine and Biomedical Sciences (ICBAS), University of Porto, 4050-313 Porto, Portugal; sergiosilva.1999@hotmail.com; 6 Epidemiology Research Unit (EPIUnit), Instituto de Saúde Pública da Universidade do Porto, 4050-600 Porto, Portugal; 7 Laboratory for Integrative and Translational Research in Population Health (ITR), 4050-600 Porto, Portugal

**Keywords:** *Cryptosporidium* spp., *Giardia duodenalis*, *Enterocytozoon bieneusi*, *Blastocystis* sp., *Balantioides coli*, PCR l

## Abstract

Enteropathogenic parasites are of significant concern for public health due to their zoonotic potential and their impact on human and animal health. In this study, we investigated their occurrence and characterized these enteropathogens in asymptomatic domestic ruminants from Portugal. A total of 302 stool samples were collected from cattle (*n* = 166), sheep (*n* = 73), and goats (*n* = 63) in various regions of Portugal and tested for *Cryptosporidium* spp., *Giardia duodenalis*, *Enterocytozoon bieneusi*, *Blastocystis* sp., and *Balantioides coli* by PCR. The occurrence of *Cryptosporidium* spp. was found to be 12.7% (8/63, 95% confidence interval [CI]: 5.65–23.5) in goats; however, no sample was found to be positive for *Cryptosporidium* spp. in cattle and sheep. For *E. bieneusi*, 6.35% (4/63; 95%CI: 1.76–15.47) of goats were found to be positive; however, no cattle or sheep were found to be positive. *Blastocystis* sp. was found in sheep (9.59%; 7/73; 95% [CI]: 0.394–18.76) and goats (12.70%; 8/63; 95% [CI]: 5.65–23.50) but none was found in cattle. No positive results for *G. duodenalis* or *B. coli* were detected in this study. This study provides essential baseline information for understanding the silent shedding and epidemiology of these enteropathogens in Portugal, contributing to overall livestock health and related occupational safety. Raising awareness among consumers, veterinarians, and farm owners is crucial to minimize the risk of transmission and promote effective disease control strategies.

## 1. Introduction

*Cryptosporidium* spp., *Giardia duodenalis*, *Enterocytozoon bieneusi*, *Blastocystis* sp., and *Balantioides coli* are prevalent pathogens known to cause diarrhea not only in humans both also in wild and domestic animals globally, including cattle and small ruminants, such as sheep and goats [1,2,3]. The fecal–oral transmission stands as the primary route of infection [4,5,6]. As such, appropriate sanitation and hygiene practices are necessary in order to effectively mitigate the spread of these protozoa and reduce the associated public health risks [7]. The protozoa can be transmitted through foodborne and waterborne routes, underscoring the importance of safe food handling and clean water sources to prevent outbreaks and infections [7]. The zoonotic potential of these pathogens is an important public health concern as it contributes to human morbidity in both developed and developing countries [4] and domestic animals such as bovine, ovine, and caprine, which are known reservoirs for a wide array of fecal protozoa [1]. 

Recent studies report the existence of 1640.65 thousand bovines, 2237.97 thousand sheep, and 350.47 thousand goats in Portugal, ranking it as the twelfth bovine and seventh ovine and caprine producers in Europe [8,9,10]. This substantial number underscores the country’s importance in the European livestock industry and agriculture but also raises alerts on the potential impact of the silent dissemination of enteric pathogens from these animals. Despite the significance of this industry, there is currently a lack of information regarding the circulation of zoonotic enteropathogenic protists, particularly *Cryptosporidium* spp., *G. duodenalis*, *E. bieneusi*, *Blastocystis* sp., and *B. coli* in Portugal. Moreover, albeit many efforts have been made to ascertain the role of these protozoa, data on the asymptomatic shedding and the role of ruminants as silent shedders remain to be fully understood, thus a better understanding the impact of this spillover to animals and humans is needed.

Cryptosporidiosis is a major cause of moderate-to-severe diarrhea in humans and has been proven to be connected to infected calves and small ruminants [11,12], underscoring the vital role of ruminants as reservoirs for this pathogen [13]. Among cattle, the prevalent *Cryptosporidium* species include *C. parvum*, *C. bovis*, *C. ryanae*, and *C. andersoni* [14], whereas in sheep and goats, the most prevalent *Crypstosporidium* species include *C. parvum*, *C. ubiquitum*, and *C. xiaoi* [12]. *Cryptosporidium* spp. are intracellular protozoan that exist outside the host cell’s cytoplasm and are recognized for inducing gastrointestinal disorders. *Cryptosporidium* follows a direct life cycle, enabling it to propagate within the gastrointestinal epithelial cells of diseased animals [15]. Human infection can occur through the consumption of minute oocyst quantities, highlighting the pathogen’s capacity for dissemination and interspecies transmission [16]. Furthermore, a challenge posed by *Cryptosporidium* pertains to its impressive resistance to conventional water treatment methodologies, including chlorination [16]. *Giardia duodenalis* is a zoonotic parasite found among calves, beef and dairy cattle, sheep, and goats [17,18,19]. The trophozoite represents the vegetative stage during which noninvasive trophozoites replicate within the mucosa of the small intestine of the host [20]. On the other hand, the cyst serves as the infective stage, with environmental resistance allowing cysts to be excreted in the host’s stool and transmitted to a new host through ingestion [21,22]. *Giardia duodenalis* exhibits eight flagella which contribute to its motility. Moreover, molecular methods have identified and categorized seven assemblages based on their genetic characteristics and host specificity [23]. *Enterocytozoon bieneusi* is a microsporidium that can be found in various hosts, including cattle and humans, highlighting its zoonotic potential [24,25], Infection can arise from either direct or indirect contact with fecal matter [26]. The progression of infection involves spores introducing the infective sporoplasm into host enterocytes via the discharged polar tube. This is succeeded by the formation of meronts, which mature into multinucleated types [27,28]. These plasmodia then undergo sporogony, leading to the creation of sporoblasts. Eventually, fully developed spores emerge, subsequently exiting the infected cells and being expelled in the stool [27]. *Blastocystis* sp. has emerged as the prevailing single-celled intestinal parasite in human populations, particularly in developing countries, where its prevalence often exceeds 50% [29]. This widespread protozoan parasite is widely acknowledged as one of the most common intestinal pathogens, regularly encountered in both humans and various animal species [30] including cattle, sheep, goat, mammals, birds, reptiles, and insects [31,32]. This unicellular, anaerobic protozoan parasite commonly resides in the large intestine, displaying a diverse life cycle that showcases its remarkable adaptability [33], and the infective cyst stage, known for its resilience, plays a pivotal role in facilitating the transmission of the parasite between hosts [34]. Conversely, recent evidence suggests a potential association between the amoeboid form of *Blastocystis* and its ability to cause disease [34]. *Balantioides coli* is the only ciliate parasite capable of infecting humans [4]. Pigs have been identified as the main reservoir for this parasite [35]. However, recent data suggest that other mammals, including cattle, sheep, and goats, can also serve as potential reservoirs for *B. coli* [4]. The life cycle of this parasite is characterized by its simplicity, commencing with the formation of dormant cysts, and its ability to replicate potentially invasive, ciliated trophozoites [3]. Upon excystation in the small intestine, ciliated, motile trophozoites are released, colonizing the lumen of the large intestine [3]. Within the large intestine, the parasite undergoes multiplication by binary fission. Both cysts and trophozoites are excreted in stool, with only cysts retaining infectivity for a new host [34].

Despite the significance of this industry, there is currently a lack of information regarding the circulation of zoonotic enteropathogenic protists, particularly on asymptomatic animals which carry particular risk for silent shedding. Therefore, the primary objective of this study was to detect and characterize the circulation of *Cryptosporidium* spp., *G. duodenalis*, *E. bieneusi*, *Blastocystis* sp., and *B. coli* in domestic asymptomatic ruminants from Portugal.

## 2. Materials and Methods

### 2.1. Sample Collection

**Figure 1:**
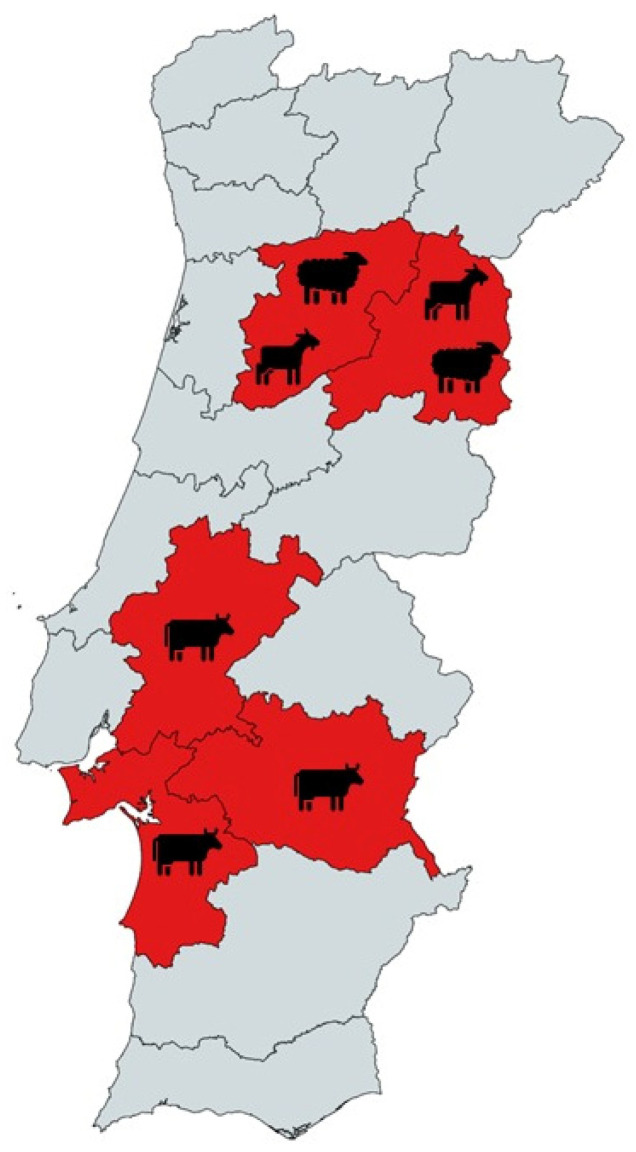
Geographical distribution of sampled animals from Portugal tested for *Cryptosporidium* spp., *Giardia duodenalis*, *Enterocytozoon bieneusi*, *Blastocystis* sp., and *Balantioides coli*.

A total of 302 individual stool samples were collected. Bovine stool samples were collected in June/July 2015 from seven farms located in Setúbal, Évora, and Santarém districts, central/southern region of Portugal (Figure 1). Of the total bovine sampled (*n* = 166), 142 were from intensive and 24 were from extensive farming systems.

Ovine stool samples were collected in two periods, June/July 2015 and March 2023. The 2015 samples (*n* = 23) were from intensive farming systems. The 2023 samples (*n* = 50) were from intensive farming systems from Viseu and Guarda (*n* = 41) and from a slaughterhouse (*n* = 9) located in the central region of Portugal. Caprine stool samples (*n* = 63) were collected also in March 2023 and from the same slaughterhouse as sheep stools. Stool samples from animals at farms were collected directly from the rectum of the animals or from freshly defecated stool. The collection of stool samples from the slaughterhouse focused on the posterior section of the large intestine. Stools were collected if they were found to have a well-structured texture and hence with no signs of gastrointestinal disease. None of the ruminants were sacrificed for the purpose of this study. All stool samples were promptly stored at 4 °C and transported to the laboratory within 12 h of collection. Upon arrival, the samples were subsequently preserved at −20 °C until the DNA extraction process, which was completed within 2 weeks after their initial collection.

### 2.2. DNA Extraction

Fecal suspensions (10%) were prepared in phosphate-buffered saline at pH 7.2 and subsequently centrifuged for 5 min at 8000× *g*. The extraction and purification of DNA was conducted simultaneously using the QIAamp Cador Pathogen Mini Kit (Qiagen, Hilden, Germany), following the manufacturer’s instructions. Specifically, 200 µL of the clarified supernatants were processed using the QIAcube^®^ automated platform (Qiagen). The eluted DNA was then appropriately stored at −80 °C with RNase-free water.

### 2.3. Molecular Detection of Cryptosporidium spp.

To identify *Cryptosporidium* spp., a nested PCR was performed, amplifying the 587-bp fragment of the ssu rRNA gene with the primer sets CR-P1/CRP2 and CR-P3/CPB-DIAGR, as described in [36].

### 2.4. Molecular Detection of Giardia duodenalis

For the detection of *G. duodenalis*, a nested PCR strategy was employed. The initial primer pair RH11-derivates and Gia2150c was used to amplify a 497-bp product, followed by a secondary primer pair RH11-derivates and RH4-derivates, amplifying a 293-bp fragment. The method was used as described in [37].

### 2.5. Molecular Detection of Enterocytozoon bieneusi

The detection of *E. bieneusi* was attempted through a nested PCR, amplifying the 390-bp fragment using the primer sets EBITS3/EBITS4 and EBITS1/EBITS2-4, as described in [38].

### 2.6. Molecular Detection of Blastocystis sp.

The detection of *Blastocystis* sp. was conducted using the method developed by [39]. This method employs a direct PCR approach that amplifies a 600-bp region of the SSU rRNA gene with the pan-*Blastocystis* barcode primer set RD5/BhRDr.

### 2.7. Molecular Detection of Balantioides coli

To attempt the detection of *B. coli*, a direct PCR was performed following the method outlined by [40]. The method targets the complete ITS1–5.8s-rRNA–ITS2 region and the last 117 bp (3’ end) of the *ssu* rRNA gene, resulting in the amplification of a 400-bp product, utilizing the primer set B5D/RD5.

### 2.8. General Procedures

Oligonucleotides used for the molecular detection of the parasites described above are shown in Table 1. 

### 2.9. Statistical Analysis

Information and first-data processing was conducted using Microsoft Office 365 Excel. Descriptive analysis was performed using IBM SPSS version 28.0.0.0 software for Windows (SPSS, Chicago, IL, USA)]. For all of the analysis, the confidence interval (CI) was established at 95%.

All endpoint, nested, and semi-nested PCR reactions were performed on a T100 thermocycler (Bio-Rad). The reaction mixtures included Fast PCR Mastermix (GriSP^®^) and 2× Xpert Fast Hotstart Mastermix (GriSP^®^). After PCR amplification, the DNA fragments were separated and visualized through electrophoresis on 1.5% agarose gels stained with Xpert Green Safe DNA gel dye (GriSP^®^). Electrophoresis was carried out at 120 V for 25 min. To confirm the results, UV light was used for irradiation of the agarose gels.

### 2.10. Sequencing and Phylogenetic Analysis

Amplicons that showed positive results and matched the expected size were purified using the GRS PCR and Gel Band Purification Kit (GriSP^®^). Following purification, the Sanger method was utilized with specific internal primers for the target gene. Bidirectional sequencing was performed, and the resulting sequences were aligned and compared to those present in the NCBI (GenBank) nucleotide database, accessed on 15 July 2023, using the BioEdit Sequence Alignment Editor v7.1.9 software package, version 2.1. Furthermore, MEGA version X software was employed for additional analysis and interpretation of the sequences [41]. The Hasegawa–Kishino–Yano model was used to estimate the ML bootstrap values using 1000 replicates. This model was determined by MEGA version X [41] to be the most effective replacement. The sequences obtained in this study were deposited in GenBank with accession numbers OR722518 (*C. parvum*), OR726049 (*C. ubiquitum*), OR726043, OR726044, OR726045, OR726046, OR726047, OR726048 (*C. xiaoi*), OR722476, OR722477, OR722478, OR722479, OR722480, OR722481, OR722482, OR722483, OR722484, OR722485, OR722486, OR722487, OR722467, OR722468, OR722469 (*Blastocystis* sp.), and OR727793, OR727794, OR727795, OR727796 (*E. bieneusi*).

## 3. Results

*Cryptosporidium* spp. was detected in 8 out of the 302 samples, with a total occurrence of 2.65% (95% confidence interval [CI]: 1.15–5.15). All *Cryptosporidium* spp. were found in goats, all retrieved from the slaughterhouse, with an occurrence of 12.7% (8/63, 95% [CI]: 5.65–23.5). BLAST analysis showed that six sequences were identified as *C. xiaoi* (99.77 to 100% identity with sequences from goat and sheep from China, KM199758, MH059800, and OL376579), one sequence as *C. parvum* (98.37% identity with a sequence from a human in The Netherlands, MH796372), and one as *C. ubiquitum* (99.55% identity with a sequence from an alpaca from China, MN876847).

*Enterocytozoon bieneusi* was detected in 4 out of the 302 samples, with a total occurrence of 1.32% (95% [CI]: 0.36–3.36). All *E. bieneusi* were exclusively found in goats, all retrieved from the slaughterhouse, with an occurrence of 6.35% (4/63, 95% [CI]: 1.76–15.47%). BLAST analyses revealed that sequences shared 95.67–100% identity with *E*. *bieneusi* sequences from animals from China (goat, fallow deer, and horse, KP262365, MT895455, and MN704934). 

*Blastocystis* sp. was detected in 15 out of the 302 samples, with a total occurrence of 4.97% (15/302 95% CI: 2.81–8.06). Seven positive samples were sheep (three retrieved from the slaughterhouse and the remaining four from intensive farms) and eight from goats, all retrieved from the slaughterhouse, with an occurrence of 9.59% (7/73 95% [CI]: 0.394–18.76) and 12.70% (8/63, 95% [CI]: 5.65–23.50), respectively. BLAST analyses of 12 sequences showed highest hits to *Blastocystis* sp., and three sequences showed highest hits to *Blastocystis hominis* detected in sheep and goats from the Czech Republic. 

In cattle, no evidence of parasitic presence was found; for goats, no evidence of *G. duodenalis* or *B. coli* was found; and for sheep, neither *E. bieneusi*, *B. coli* nor *Cryptosporidium* spp. were detected. 

**Figure 2:**
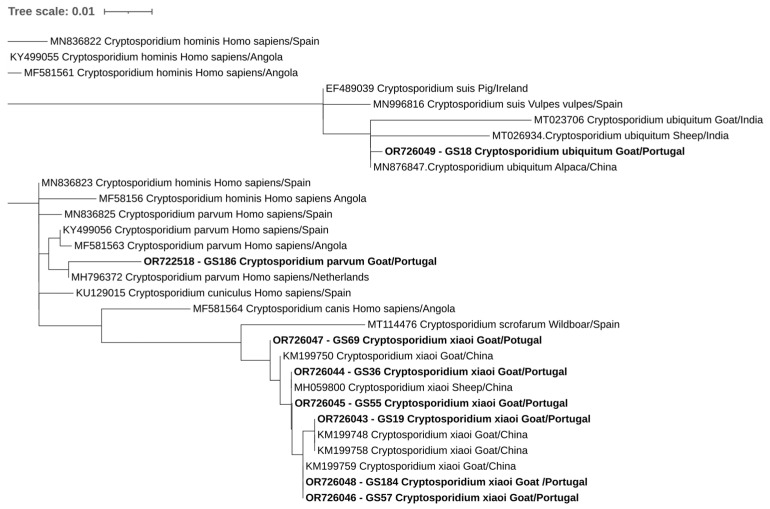
Phylogenetic analysis of *Cryptosporidium* found in ruminants. Tree was inferred using the MEGA X maximum likelihood method (Tamura 3-parameter model) and the Interactive Tree of Life (iTOL). The samples in this study are in shown in bold and plus 22 strains of different *Cryptosporidium* species obtained from GenBank are shown without bold and are identified with the accession number and its species and country of origin.

**Figure 3:**
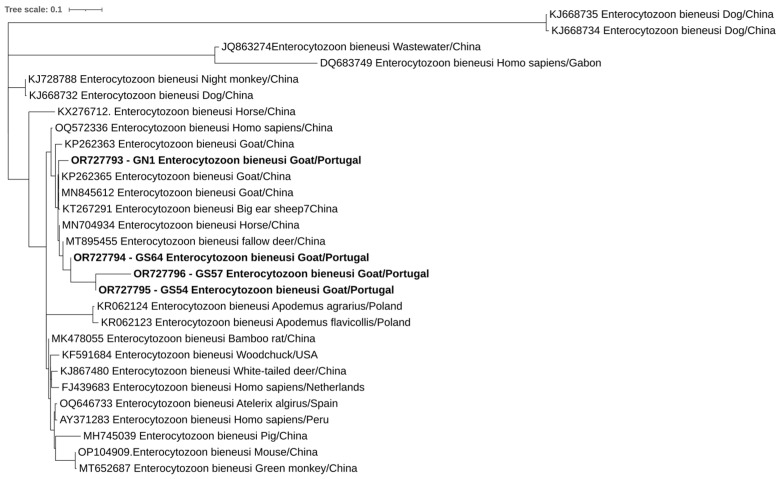
Phylogenetic analysis of *E. bieneusi* found in ruminants. Tree was inferred using the MEGA X maximum likelihood method (Hasegawa–Kishino–Yano) and the Interactive Tree of Life (iTOL). The samples in this study are in shown in bold and 25 strains of different Enterocytozoon species obtained from GenBank are shown without bold and are identified with the accession number and its species and country of origin.

**Figure 4:**
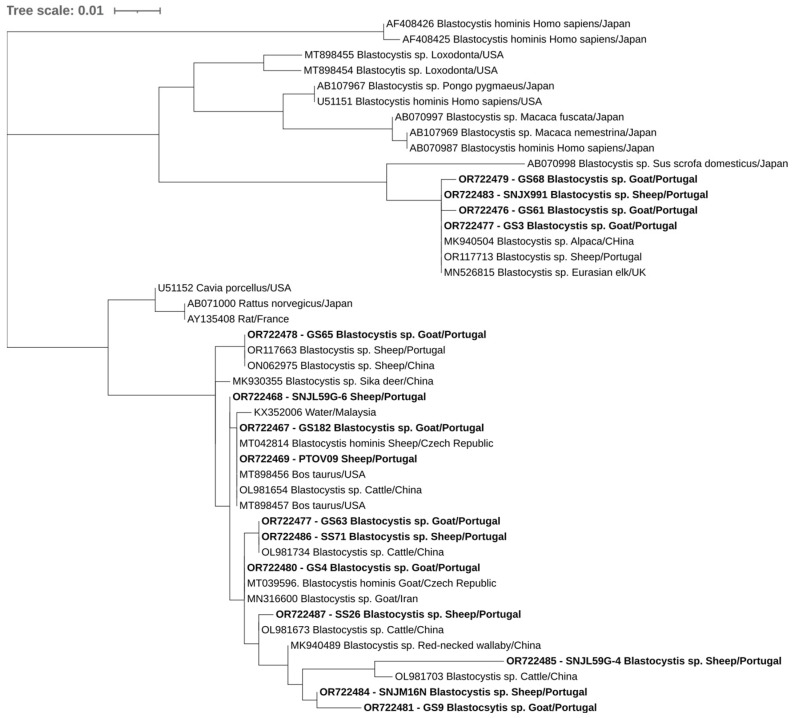
Phylogenetic analysis of *Blastocystis* sp. found in ruminants. Tree was inferred using the MEGA X maximum likelihood method (Tamura 3-parameter model) and the Interactive Tree of Life (iTOL). The samples in this study are in shown in bold and 30 strains of different *Blastocystis* species obtained from GenBank are shown without bold and are identified with the accession number and its species and country of origin.

Overall, at least 26 of the 302 animals in this study were positive for at least one of the studied protozoa. One of the four goats positive for *E. bieneusi* was also positive for *C. xiaoi*. Phylogenetic trees inferred for *Cryptosporidium* spp., *E. bieneusi*, and *Blastocystis* sp. sequences confirmed the classifications via BLAST (Figure 2, Figure 3 and Figure 4).

## 4. Discussion

In this study, we report the occurrence and phylogenetic characterization of *Cryptosporidium* spp., *E. bieneusi,* and *Blastocystis* sp. in Portuguese ruminants via molecular methods. *Cryptosporidium* was exclusively found in goats within this sampling, resulting in an occurrence of 12.7% (8/63, 95% [CI]: 5.65–23.5). We found three *Cryptosporidium* spp., namely, *C. parvum*, *C. ubiquitum,* and *C. xiaoi*, the last two identified for the first time in Portugal, with occurrences of 1.59% (1/63, 95% [CI]: 0.04–8.53) and 9.52% (6/63, 95% [CI]: 3.58–19.59), respectively. Additionally, *C. parvum* was found in 1.59% (1/63, 95% [CI]: 0.04–8.53) of the goats. These results are particularly intriguing since a recent review on small ruminants and zoonotic cryptosporidiosis showed that in Europe, *C. parvum* is the most frequently encountered species in goats and sheep, accounting for 62.5% of reported cases, while *C. xiaoi* represents 19.3% and *C. ubiquitum* 12.2% [12]. The identification of *C. ubiquitum* and *C. parvum* holds relevance as they represent zoonotic agents, while *C. xiaoi* appears to be primarily adapted to ovine and caprine [42].

Comparing our findings with other studies on the same host, the present study found 12.7% of goats to be shedding *Cryptosporidium* spp., a number notably lower than that reported in other European countries, with 65.7% in Greece [43], 53.3% in France [44], and 19.1% in Spain [45]. Alerts should be made to the fact that different detection methods (PCR, microscopic) were used in the different studies, yielding distinct sensitivities and specificities. Moreover, even when comparing studies also using molecular biology tools, distinct nucleic acid extraction methods and even the use of different oligonucleotide primers which target different genomic regions could have alone or combined produced results with different sensitivities.

*Enterocytozoon bieneusi* was only found in goats, occurring in 6.35% (4/63, 95% [CI]: 1.76–15.47) of these animals. *Enterocytozoon bieneusi* is one of the most frequently identified microsporidia in humans and domestic as well as wild animals worldwide [46], responsible for causing 90% of human microsporidiosis [47]. The presence of *E. bieneusi* is of particular concern due to its zoonotic potential, posing a threat to both human and livestock health [27]. 

In the present study, the occurrence of *E. bieneusi* was lower than that reported in goats from Thailand (19.2%) [48], China (21.8%) [49], and Tibet (9.6%%) [50].

In this study, *Blastocystis* sp. was found in both sheep and goats, occurring in 9.59% and 12.70% of these animals, respectively. In comparison to the available data, the occurrence in sheep of 9.59% (7/73, 95% [CI]: 3.94–18.76) was lower than that reported in Greenland (14%), Iran (32.0%), Turkey (38.2%), and China (16.26%) [51,52,53,54]. Interestingly, the prevalence of *Blastocystis* sp. in sheep remains relatively understudied in Europe. Also, interestingly, all *Blastocystis* sequences from sheep were classified as *Blastocystis* sp. In the present study, goats presented a higher occurrence of *Blastocystis* than sheep, 12.70% (8/63, 95% [CI]: 5.65–23.56), with all of cases being classified as *Blastocystis* sp. In this study, *Blastocystis* occurrence in goats was again lower when compared to Malaysia (30.9%) [55], Italy (44.4%) [56], and China (33.63%) [57]. This could be due not only to differences in diagnostic performances but also to genetic and eco-epidemiological differences between sampled animals which could ultimately influence the circulation of this agent. Noteworthy, animals can serve as reservoirs for *Blastocystis* sp. due to its poor host specificity and zoonotic potential. This parasite is commonly detected in fecal samples from both animals and humans worldwide and is estimated to cause nearly one billion human infections, making it one of the most commonly found parasites in humans [31,52,58]. Despite our findings, one cannot exclude the possibility of these numbers underrepresenting the true scenario of these enteropathogenic agents circulating in healthy domestic ruminants in Portugal. When analyzing stool samples, care should be taken since PCR inhibitors can be found, such as phenolic compounds, glycogen, fats, cellulose, constituents of bacterial cells, nontarget nucleic acids, and heavy metals, leading to false negatives [59].

Although the results of this study indicate the absence of certain gastrointestinal parasites in each animal—cattle (absence of *Cryptosporidium* spp., *Blastocystis* sp., *E. bieneusi*, *G. duodenalis*, and *B. coli*), sheep (absence of *Cryptosporidium* sp., *G. duodenalis*, and *B. coli*), and goats (absence of *B. duodenalis* and *B. coli*)—other studies have reported the presence of these parasites in these animals. *Cryptosporidium* spp. has been found in cattle in Portugal (17.6%), Spain (16.7%), Italy (10,1%), Poland (17%), and the UK (10.2%) [57,60,61,62,63]. *Blastocystis* sp. has been found in cattle with an occurrence of 32.1% in Spain, 6.7% in Korea, 19.4% in Egypt, and 10.3% in China [30,64,65,66]. *Enterocytozoon bieneusi* has been found in cattle with an occurrence of 6.25% in Portugal, 0.6% in Spain, 28.3% Thailand, 17.5% in Brazil, and 4.4% in the USA [48,64,67,68,69]. As for *G. duodenalis,* the parasite has also been found in cattle with an occurrence of 9% in Portugal, 18.8% in Spain, 34.3% in the UK, and 8% in China [70,71,72,73]. *Balantidoides coli* is a rare finding in cattle. To the authors’ best knowledge, the only study on *B. coli* in cattle showed a prevalence of 25% in Pakistan [74].

As for sheep, the prevalence of 10.1% of *Cryptosporidium* spp. has been observed in Italy, 12.6% in Madagascar, and 12.3% in Tibet. *Giardia duodenalis* in sheep was found with prevalences of 30% in Iran, 3.6% Turkey, and 37.3% Greece. Regarding *B. coli*, prevalences of 7.9% in Bangladesh, 3.99% in Pakistan, 1.99% in Egypt, and 1% in Romania [75,76,77] have been shown. It is worth noting that there are limited data available on the prevalence of *B. coli* in sheep, indicating the need for further research in this area. The prevalence of *E. bieneusi* in sheep has been reported in Sweden (45.0%), Brazil (19.2%), and Slovakia (4.4%) [78,79,80].

For goats, the presence of *G. duodenalis* has been observed in Spain (7.7%), 5.43% in China, 40.4% in Greece, and 5% in Iran [81,82,83,84]. *Balantioides coli* in goats was found with prevalences of 4% in Egypt, 7.75% in Nepal, 3% in Kenya, 4.8% in Tanzania, and 3.46% in Pakistan [76,85,86,87,88]. Since goats do not seem to be natural hosts for this parasite, there is still a lack of data about their prevalence. 

In this study, the results suggest that within Portuguese farming systems, healthy ruminants pose limited zoonotic impact as reservoirs for enteropathogenic parasites. Unlike previous studies on the topic which have used microscopy to ascertain the circulation of fecal protozoa in ruminants, [1] we used molecular biology tools such as PCR and nested PCR, known for their superior sensitivity and specificity. Hence, despite the low occurrence found in the present study, the likelihood for false positives is low.

Overall, at least 26 of the 302 animals in this study were positive for at least one of the studied protozoa. Interestingly, the majority of the stools positive for protozoa were retrieved from the slaughterhouse processing facility, and this likely represents the highest risk of transmission from ruminants to humans. Factors such as disparities in animal management practices, variations in sample sizes utilized in research, local climatic and environmental conditions, potential contamination of feed and water sources, and the overall health and immune status of the animals may alone or in combination influence the data shown here. 

## 5. Conclusions

The prevalence of gastrointestinal parasites in Portuguese ruminants (cattle, sheep, and goats) is relatively low, suggesting a limited role as enteropathogenic protist reservoirs. However, silent shedding of zoonotic parasites like *C. parvum*, *C. ubiquitum*, *E. bieneusi,* and *Blastocystis* sp. was found, thus requiring ongoing surveillance. These findings provide a baseline for understanding parasite epidemiology and improving livestock and food safety. Preventing pathogen transmission requires a comprehensive approach with biosecurity and raising awareness among consumers, veterinarians, and farm owners, which can mitigate the impact on human and animal health. The use of sensitive detection methods and regular surveillance will be essential for monitoring any changes in parasite prevalence over time and developing effective disease control strategies for Portuguese ruminants.

## Figures and Tables

**Table 1:** Oligonucleotides used for the molecular detection of enteropathogenic protists in asymptomatic domestic ruminants from Portugal.

Target Organism	Locus	Oligonucleotide	Sequence (5′–3′)	Reference
*Blastocystis* sp.	*ssu* rRNA	RD5	GAGCTTTTTAACTGCAACAACG	[39]
		BhRDr	ATCTGGTTGATCCTGCCAGT	
*Cryptosporidium* spp.	*ssu* rRNA	CR-P1	CAGGGAGGTAGTGACAAGAA	[36]
		CRP2	TCAGCCTTGCGACCATACTC	
		CR-P3	ATTGGAGGGCAAGTCTGGTG	
		CPB-DIAGR	TAAGGTGCTGAAGGAGTAAGG	
*Giardia duodenalis*	*ssu* rRNA	RH11	CATCCGGTCGAT CCT GCC and CAT CCG GTT GAT CCT GCC	[37]
		Gia2150c	CTGCTGCCGTCCTTGGATGT	
		RH4	AGTCGAACCCTGATTCTCCGCCAGG and AGTCAAACCCTGATCCTCCGCCAGG and AGTCGAACCCTGATTCTCCGTCAGG	
*Enterocytozoon bieneusi*	ITS	EBITS3	GGTCATAGGGATGAAGAG	[38]
		EBITS4	TTCGAGTTCTTTCGCGCTC	
		EBITS1	GCTCTGAATATCTATGGCT	
		EBITS2.4	ATCGCCGACGGATCCAAGTG	
*Balantioides coli*	ITS	B5D	GAGCTTTTTAACTGCAACAACG	[40]
		RD5	ATCTGGTTGATCCTGCCAGT	

## Data Availability

The data presented in this study are available on request from the corresponding author.
